# Using Machine Learning Algorithms to Predict Hepatitis B Surface Antigen Seroclearance

**DOI:** 10.1155/2019/6915850

**Published:** 2019-06-11

**Authors:** Xiaolu Tian, Yutian Chong, Yutao Huang, Pi Guo, Mengjie Li, Wangjian Zhang, Zhicheng Du, Xiangyong Li, Yuantao Hao

**Affiliations:** ^1^Department of Medical Statistics and Epidemiology & Health Information Research Center & Guangdong Key Laboratory of Medicine, School of Public Health, Sun Yat-sen University, Guangzhou 510080, China; ^2^Department of Infectious Diseases, The Third Affiliated Hospital, Sun Yat-sen University, Guangzhou 510630, China; ^3^School of Data and Computer Science, Sun Yat-sen University, Guangzhou 510006, China; ^4^Department of Public Health, Medical College of Shantou University, Shantou 515063, China; ^5^Department of Environmental Health Sciences, School of Public Health, University at Albany, State University of New York, Rensselaer 12144, USA; ^6^Sun Yat-sen Global Health Institute, Sun Yat-sen University, Guangzhou 510080, China

## Abstract

Hepatitis B surface antigen (HBsAg) seroclearance during treatment is associated with a better prognosis among patients with chronic hepatitis B (CHB). Significant gaps remain in our understanding on how to predict HBsAg seroclearance accurately and efficiently based on obtainable clinical information. This study aimed to identify the optimal model to predict HBsAg seroclearance. We obtained the laboratory and demographic information for 2,235 patients with CHB from the South China Hepatitis Monitoring and Administration (SCHEMA) cohort. HBsAg seroclearance occurred in 106 patients in total. We developed models based on four algorithms, including the extreme gradient boosting (XGBoost), random forest (RF), decision tree (DCT), and logistic regression (LR). The optimal model was identified by the area under the receiver operating characteristic curve (AUC). The AUCs for XGBoost, RF, DCT, and LR models were 0.891, 0.829, 0.619, and 0.680, respectively, with XGBoost showing the best predictive performance. The variable importance plot of the XGBoost model indicated that the level of HBsAg was of high importance followed by age and the level of hepatitis B virus (HBV) DNA. Machine learning algorithms, especially XGBoost, have appropriate performance in predicting HBsAg seroclearance. The results showed the potential of machine learning algorithms for predicting HBsAg seroclearance utilizing obtainable clinical data.

## 1. Introduction

HBV infection remains an urgent public health issue worldwide. Roughly 257 million individuals have been infected with HBV, and more than 350 million patients are living with CHB [1]. It is well documented that HBsAg seroclearance is an important milestone for prognosis during the treatment of CHB [2–4]. The annual incidence of spontaneous HBsAg seroclearance in chronically HBV-infected patients varied from 0.45% to 2.38% worldwide, indicating that HBsAg seroclearance is a rare event [5–10]. Previous studies have suggested a potential association between spontaneous or therapy-induced HBsAg seroclearance and a better prognosis, liver histological improvement, a diminished risk of hepatocellular carcinoma (HCC), and extended survival [11–13]. Therefore, HBsAg seroclearance is an important endpoint achieving a better outcome of antiviral therapy.

Evidences on relevant viral factors and host characteristics of HBsAg seroclearance have been reported in previous studies. Researchers have investigated that low serum HBsAg levels alone or joined up with a low serum HBV DNA load were important determinants of HBsAg seroclearance [8, 14]. As for the host characteristics, age is one of the most important characteristics in HBsAg seroconversion, followed by factors of gender, fatty liver, cirrhosis at baseline or developed during follow-up, and alanine aminotransferase (ALT) levels at baseline [6]. However, previous studies developing prediction models were mainly based on long-term tracking of limited factors and traditional statistical methods, of which the estimates maybe biased due to the potential collinearity issue for high-dimensional medical data. To address the knowledge gap, in this study, we used machine learning algorithms instead of traditional models to determine the association between obtainable clinical variables and HBsAg seroclearance. Machine learning algorithms have attracted considerable attention in health domain in recent years. It has been successfully applied as powerful classification methods to extract effective information from the high-dimensional, correlated, nonlinear, and imbalanced clinical datasets and make accurate diagnosis and predictions decisions [15, 16]. However, no existing models have been identified to achieve the best performance for HBsAg seroclearance prediction. In this study, we generated multiple appropriate machine learning models including XGBoost, RF, DCT, and LR according to the characteristics of the dataset (highly dimensional and imbalanced) and aimed to identify the optimal one. The main purpose of this study is to identify the optimal machine learning model for predicting the HBsAg seroclearance in a retrospective cohort of patients with CHB.

## 2. Materials and Methods

This study included chronic hepatitis B patients enrolled into the SCHEMA cohort (South China Hepatitis Monitoring and Administration cohort) between January 2006 and June 2015. Each patient was diagnosed following the “Guideline: prevention and treatment of viral hepatitis” revised in 2010 and followed up by staff in the Infectious Diseases Department of the Third Affiliated Hospital, Sun Yat-sen University. For the current study, we excluded patients who met at least one of the following conditions: (1) lost follow-up for over 6 months; (2) had an HBV DNA baseline under detection; (3) received interferon treatment previously; (4) developed comorbidities such as hepatitis A/C/E virus infection, decompensated liver disease, autoimmune liver diseases, malignant tumors, and renal insufficiency; and (5) received immunosuppressive (transplantation) therapy. There were 2235 CHB patients included in this study.

The endpoint (HBsAg seroclearance) was defined as loss of HBsAg detectability during follow-up by the qualification method using ECL kits (Roche Laboratories, Germany; lower limit of detection (LLOD), 0.05 IU/ml). We collected the following information for each patients: age, gender, BMI (body mass index), drinking history, family history, diagnosis of the disease phase, treatment (including lamivudine (LAM), telbivudine (LDT), entecavir (ETV), adefovir (ADV), and tenofovir (TDF) and the changing times of treatment was recorded as lines), virological response after treatments, routine pathology measurements, and other clinical measurements. Regular follow-ups were performed every 1–3 months. Thirty features including laboratory tests, clinical manifestations, and drug treatment strategies were recorded at baseline before the occurrence of HBsAg seroclearance. Verbal informed consent was obtained for all participants upon their first and subsequent follow-up visits.

Results of routine liver biochemical function tests were also obtained including serum levels of alanine aminotransferase (ALT), aspartate aminotransferase (AST), serum albumin (ALB), gamma-glutamyl transferase (GGT), total bilirubin (Tbil), direct bilirubin (Dbil), as well as a range of erythrocyte and leucocyte markers (hemoglobin (Hb), platelet (PLT) count, and white blood cell (WBC) count). The measurements were performed on Autobiochemical Analyzer (7600-020; HITACHI, Tokyo, Japan) following a standard protocol.

Serum HBsAg and hepatitis B virus E antigen (HBeAg) were both measured quantitatively by Elecsys kits (Roche Laboratories, Germany). Serum HBV DNA level was measured using the Cobas TaqMan HBV RT-PCR test (CAP-CTM; Roche Molecular Systems).

Radiological indicators including right liver oblique diameter, spleen portal width, spleen length, and spleen portal vein width were measured to reflect the thickness and width of patients' liver and spleen.

A total of 30 variables were included in the dataset. Ten of them are categorical variables including gender, drinking history, HBV family history, initial diagnosis, current diagnosis, lines (number of treatment replacements), initial treatment, current treatment, and virological response. The rest are continuous variables including age, BMI, serum indicators, and radiological indicators. The dataset was split into the training dataset (70%) and test dataset (30%) to train and test the machine learning models. The training set contains a known output, and the model learns with these data in order to be generalized to new data. The test size was 0.30, indicating that 30% of the data were withheld for testing.

In this study, the predictive models were built based on four machine learning classification algorithms: logistic regression, decision tree, random forest, and extreme gradient boosting by using Python programming software with version 3.6. The generations of each model for predictions required tuning of several key parameters. The value of each parameter was chosen by using grid search and 5-fold (stratified K-fold) cross-validation, with the training dataset split into 5 equal size subsets randomly for five times of cross-validation. Each round of cross-validation involved a process of performing the model generation on four subsets (as the development set) and a process of validating on the remaining subset (as the validation set). For evaluation purpose, metrics including areas under the receiver operating characteristic curves (AUCs) of the models, *F* score, confusion matrix, precision, and recall are calculated by 5-fold (stratified K-fold) cross-validation. *F* score represents the harmonic mean of precision and recall. Precision represents the percentage of tuples that the classifier has labeled as positive is actually positive. Sensitivity represents the true-positive recognition rate:

(1) $$\begin{array}{c} F‐\text{score}=\frac{2\;*\text{ TP}}{2\;*\text{ TP}+\text{FP}+\text{FN}},\\ \text{Precision}=\frac{\text{TP}}{\text{TP}+\text{FP}},\\ \text{Sensitivity}=\frac{\text{TP}}{\text{TP}+\text{FN}}. \end{array}$$

where true positive (TP) represents positive case correctly identified as positive, false positive (FP) represents negative case incorrectly identified as positive, and false negative (FN) represents positive people incorrectly identified as negative.

Logistic regression model is a classic statistical classification method developed in 1958 by David Cox which is widely used for modelling binary-dependent variable and is now very commonly used in scientific study, including biology, medicine, health, and clinical research [17]. Logistic regression investigates the correlation between binary-dependent variable and -independent variables by estimating probabilities using a logistic function, which is the cumulative logistic distribution.

Decision tree is a nonparametric supervised learning method used for classification and regression that uses a tree-like graph or model of decision to predict the value of a target variable by learning simple decision rules inferred from the data features. It can handle both numerical and categorical data, and nonlinear relationships between parameters do not affect tree performance.

Random forest model is a powerful bagging and ensemble learning method for classification and regression tasks and can provide the relative importance of the input variables by comprising multiple decision trees at training set and predictive values of classification and regression trees [18]. Random forest is one of the most accurate algorithms by averaging votes of multiple deep decision trees from different random subsets of the training set to reduce the variance. The main limitation of random forest is that a large amount of trees can make the algorithm slow and ineffective for real-time predictions.

Extreme gradient boosting was initially raised as a terminal application in a research project by Tianqi Chen which could be configured using a LibSVM configuration file [19]. Comparing with other machine learning models, XGBoost algorithms were designed to be highly efficient and flexible and are of impressive predictive accuracy. XGBoost implements a scalable parallel classification and regression trees (CART) boosting system under the Gradient Boosting framework which can widely solve data science problems in a fast and accurate way [20]. Gradient Boosting is a boosting learning algorithm which combines the estimates of a set of simpler and weaker models. Because XGBoost internally provides hyperparameters for cross-validation, regularization, user-defined objective functions, tree parameters, scikit-learn compatible API, and so on, it usually has better fitness than other models, especially in solving different types of datasets or distributions.

## 3. Results

Among 2235 CHB patients, a total of 106 patients had been identified as HBsAg seroclearance. The summary of participant's characteristics including demographic characteristics and laboratory measurements for patients is shown in Table 1. The mean age of the patients was 40.58 ± 12.07 years, and 73.2% patients were male.

The whole dataset was randomly partitioned into 1564 instances of the training set and 671 instances of the testing set measured by use of a 70%/30% split of the data.  Table 2 shows the tuning parameters and values of the final models.

**Figure 1:**
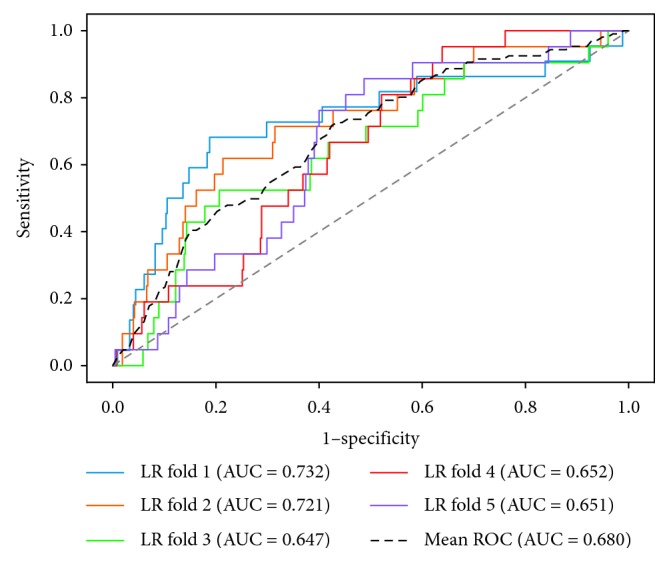
Receiver operating characteristic curves of logistic regression.

**Figure 2:**
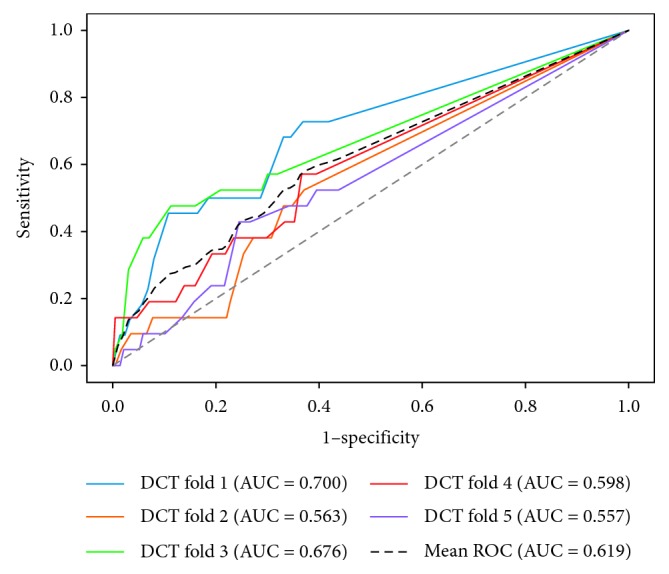
Receiver operating characteristic curves of decision tree.

**Figure 3:**
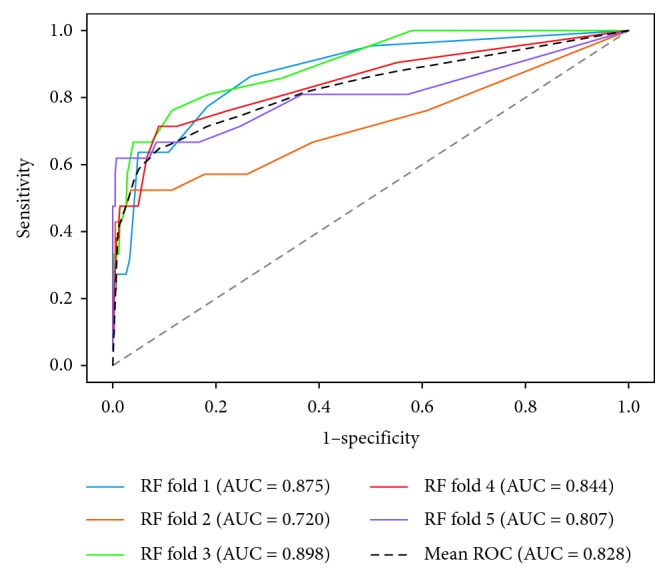
Receiver operating characteristic curves of random forest.

**Figure 4:**
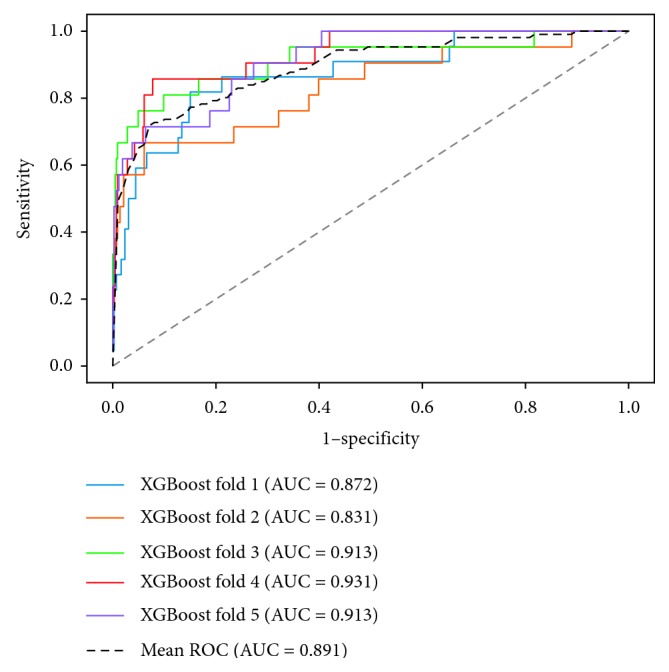
Receiver operating characteristic curves of extreme gradient boosting.

**Figure 5:**
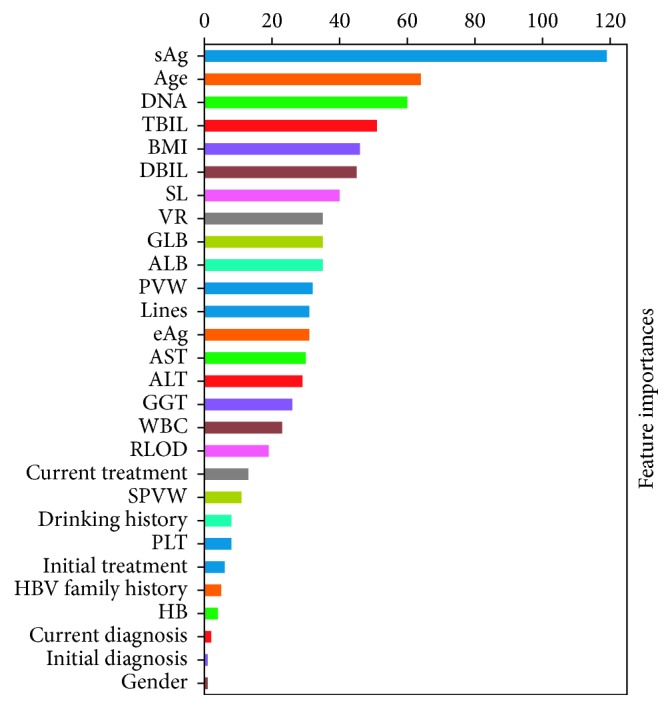
Variable importance plot of the XGBoost model for predicting HBsAg seroclearance.

The performances of four models are shown in Table 3, and the receiver operating characteristic (ROC) curves for each model are shown in Figures 1–4. The AUCs reflecting the total discriminative abilities of the XGBoost, RF, DCT, and LR were 0.891 (95% confidence interval (CI): 0.889, 0.895), 0.829 (95% CI: 0.824, 0.834), 0.619 (95% CI: 0.614, 0.624), and 0.680 (95% CI: 0.677, 0.683), respectively. XGBoost model exhibited the best AUC, and the performance was significantly better than the rest models. In terms of other measures, the *F* scores of the XGBoost, RF, DCT, and LR were 0.97, 0.97, 0.95, and 0.97, respectively. Using the variables exhibiting the highest coefficients of permutation importance for HBsAg seroclearance in the XGBoost model, the variable importance plot suggested that the level of HBsAg was the most important predictor of HBsAg seroclearance followed by age and DNA (Figure 5). The confusion matrix showed that logistic regression was severely influenced by the high degree of imbalance of the dataset, as it classified the whole sample to the negative class.

## 4. Discussion

HBsAg seroclearance has been widely considered as one of the most important indicators of CHB prognosis. Using machine learning algorithms to predict disease status or outcomes with clinical datasets is consistently gaining increasing attention in medical and health field, as shown by many previous studies inspecting relevant topics. In this retrospective cohort study, we evaluated the performance of four prediction models generated by utilizing obtainable baseline clinical data fitted with machine learning algorithms to accurately classify individuals who were likely to result in HBsAg seroclearance, with no need to acquire longitudinal data. It is of remarkable significance that, in this study, we have investigated the optimisation for machine learning algorithms of routine clinical datasets. Our results indicated the best performing prediction model-XGBoost obtained an AUC of 0.891, indicating a good prediction accuracy. The result of the serum HBsAg level acting as the most important variable shown in our study is consistent with previous study [8, 14]. Following factors such as age and serum level of DNA were also proven highly related with HBsAg seroclearance. As there is not enough evidence in this domain, our findings will help achieve targets of early prediction and detection by laboratory alternatives and assist the hepatologists in choosing the optimal therapeutic regimen.

A model using serum quantitative HBsAg (qHBsAg) and HBV DNA levels as proven clinical parameters to predict HBsAg seroclearance and seroconversion has been developed previously with artificial neural networks (ANNs), which is the only existing model on HBsAg seroclearance patients according to the best of our knowledge [21]. However, some limitations should be noted in this study including the limitation of small datasets, requirement of longitudinal follow-up data, and limited information considered except the currently proven predictor qHBsAg, an appropriate model with sufficient accuracy and generalizability for early predicting HBsAg seroconversion remains to be provided. Since deep learning architectures have several characteristic such as being more adaptive to big datasets, more likely to overfit, cost more computational work, and have more difficulties during practical implement by clinical experts, we did not generate deep learning models. To the best of our knowledge, this may be the first study utilizing machine learning algorithms to identify patients with higher probability developing to HBsAg seroclearance.

Our study had several limitations. First, as the features we included in our model were based on the datasets we obtained, unknown potentially relevant features may have been unfortunately missed. Second, the models in our study were developed using the dataset related to HBsAg seroclearance, which may not be suitable for direct application for prediction or diagnosis for other status or diseases. Third, in this study we only included four machine learning algorithms, and further exploration on investigating better models are still urgent to improve the prediction accuracy. Finally, the external applicability of our findings might be limited due to the dataset from a single center within the specific geographic region, resulting in the limitation of the sample's representativeness of the whole study population, and it may be controversial that the results might change from other centers.

## 5. Conclusions

In this study, we conducted an evaluation and comparison of four well-known machine learning algorithms by regressing the HBsAg seroclearance status of each patient against laboratory and demographic variables. The results show that machine learning algorithms, especially XGBoost, can predict HBsAg seroclearance with an efficient accuracy. This study also showed a potential of machine learning algorithms being used for clinical outcome predictions.

## Figures and Tables

**Table 1:** Summary of participant's characteristics.

Variables	Value
Age (years)^a^	40.58 ± 12.07
Gender (male)^b^	1636 (73.2)
BMI^a^	22.53 ± 3.96
Drinking history^b^	256 (11.5)
HBV family history^b^	1350 (60.4)
HCC family history^b^	188 (8.4)
Initial diagnosis^b^	
Inactive hepatitis B virus carrier	12 (0.5)
Chronic hepatitis B	1966 (88.0)
Hepatitis cirrhosis	216 (9.7)
Hepatocellular carcinoma	41 (1.8)
Current diagnosis^b^	
Hepatitis B virus carrier	13 (0.5)
Chronic hepatitis B	1875 (83.9)
Hepatitis cirrhosis	222 (9.9)
Hepatocellular carcinoma	125 (5.6)
ALT^a^ (U/L)	95.91 ± 167.82
AST^a^ (U/L)	144.68 ± 280.77
GGT^a^ (U/L)	59.32 ± 79.35
PLT^a^ (U/L)	175.19 ± 67.53
ALB^a^ (g/L)	44.61 ± 5.37
TBIL^a^ (*μ*mol/L)	25.40 ± 52.83
DBIL^a^ (*μ*mol/L)	11.17 ± 41.19
PLT^a^ (×10^9^/L)	175.19 ± 67.53
DNA^a^ (log/IU/mL)	5.57 ± 2.05
sAg^a^ (log/IU/mL)	3.42 ± 0.81
eAg^a^ (log/IU/mL)	0.71 ± 1.63
WBC^a^ (×10^9^/L)	6.03 ± 1.93
HB^a^ (g/L)	140.66 ± 34.48
RLOD^a^ (mm)	114.85 ± 27.92
PVW^a^ (mm)	11.37 ± 4.17
SL^a^ (mm)	102.74 ± 21.19
SPVW^a^ (mm)	6.17 ± 4.39
Initial treatment^b^	
None	874 (39.1)
LMV	248 (11.1)
ADV	277 (12.4)
LdT	111 (5.0)
ETV	610 (27.3)
TDF	62 (2.8)
LMV + ADV	47 (2.1)
LdT + ADV	4 (0.2)
ETV + ADV	2 (0.1)
Lines^b^	
0	1035 (46.3)
1	818 (36.6)
2	211 (9.4)
3	93 (4.2)
4	46 (2.1)
5	18 (0.8)
6	10 (0.5)
7	1 (0.0)
8	2 (0.1)
9	1 (0.0)
Current treatment^b^	
None	1019 (45.6)
LMV	68 (3.0)
ADV	152 (6.8)
LdT	26 (1.2)
ETV	61 (27.4)
TDF	252 (11.3)
LMV + ADV	79 (3.5)
LdT + ADV	6 (0.3)
ETV + ADV	21 (0.9)
IFN^b^	115 (5.1)
VR^b^	
IVR	332 (14.9)
EVR	976 (43.7)
SOR	976 (41.5)

^a^Mean and standard deviation; ^b^frequencies and percentages; VR: virological response; IVR: initial virological response; EVR: early virological response; SOR: suboptimal virological response.

**Table 2:** Summary of parameter values in each model for predicting HBsAg seroclearance.

Model	Parameter	Value
Extreme gradient boosting	n_estimators	153
	max_depth	4
	min_child_weight	2
	Subsample	0.5
	colsample_bytree	0.8
	colsample_bylevel	0.8
	reg_alpha	2.0
	reg_lambda	0.3

Random forest	max_features	Auto
	min_samples_leaf	1
	n_estimators	40

Decision tree	max_depth	29
	max_features	log2
	min_samples_leaf	23

Logistic regression	C	0.001
	Penalty	L1

**Table 3:** Summary of predictive performance of each model.

Model	TP	FN	TN	FP	Precision	Sensitivity	*F*-score	AUC (95% CI)
Logistic regression	0	35	636	0	1.00	0.95	0.97	0.680 (0.677, 0.683)
Decision tree	4	31	627	9	0.97	0.94	0.95	0.619 (0.614, 0.624)
Random forest	4	31	635	1	0.99	0.95	0.97	0.829 (0.824, 0.834)
Extreme gradient boosting	9	26	632	4	0.98	0.96	0.97	0.891 (0.889, 0.895)

## Data Availability

The data used to support the findings of this study were supplied by Xiangyong Li under license and so cannot be made freely available. Requests for access to these data should be made to [Xiangyong Li, lxy2005123@126.com].
